# Genetic Diversity and Population Differentiation of Yangtze Finless Porpoise in Poyang Lake

**DOI:** 10.3390/ani15131838

**Published:** 2025-06-21

**Authors:** Han Zhang, Denghua Yin, Jianglong Que, Xiaoyan Zhu, Danqing Lin, Congping Ying, Jinxiang Yu, Kai Liu

**Affiliations:** 1 National Demonstration Center for Experimental Fisheries Science Education, Shanghai Ocean University, Shanghai 201306, China; hanzhang0221@163.com; 2 Key Laboratory of Freshwater Fisheries and Germplasm Resources Utilization, Ministry of Agriculture and Rural Affairs, Freshwater Fisheries Research Center, Chinese Academy of Fishery Sciences, Wuxi 214081, China; yindenghua@ffrc.cn (D.Y.); lindq@ffrc.cn (D.L.); yingcongping@ffrc.cn (C.Y.); 3 Aquatic Conservation and Rescue Center of Jiangxi Province, Nanchang 330096, China; que_jianglong@sina.com; 4 Anqing Aquatic Technology Promotion Center Station, Anqing 246000, China; matthew956@163.com

**Keywords:** Yangtze finless porpoise, Poyang Lake, genetic diversity, genetic differentiation, microsatellite DNA, mitochondrial DNA

## Abstract

The Yangtze finless porpoise (*Neophocaena asiaeorientalis asiaeorientalis*; YFP) is the only freshwater cetacean species that remains in the Yangtze River, China. Poyang Lake is connected to the main stream of the Yangtze River, and the number of YFPs in Poyang Lake constitutes approximately half of the total species population. To implement effective conservation measures and formulate scientific genetic management strategies for the YFPs in Poyang Lake, we conducted population genetic analyses on 125 blood samples from the Poyang population and 46 tissue samples from the Anqing population, utilizing mitochondrial DNA D-*loop* and microsatellite loci. The genetic diversity analysis revealed two haplotypes in the Poyang population, with mitochondrial genetic diversity indices of Hd = 0.481 ± 0.020 and Pi = 0.00078 ± 0.00030. Microsatellite markers further demonstrated indices of Ho = 0.610 and He = 0.655. The genetic differentiation analysis indicated that the two populations exhibited moderate genetic differentiation (0.05 < Fst < 0.15). Upon excluding the dead samples from the Anqing population, the genetic differentiation between the two populations increased and the gene flow diminished. This indicated that certain dead samples from the Anqing population might have originated from Poyang Lake or had a background of Poyang Lake–Yangtze River migration and gene exchange. This finding was further corroborated by STRUCTURE analysis, which revealed genetic admixture between the two populations. We assessed the current genetic diversity of the Poyang population and its genetic differentiation from the Anqing population. This study provides fundamental data for formulating a conservation program for YFPs in Poyang Lake.

## Simple Summary

The Yangtze finless porpoise (*Neophocaena asiaeorientalis asiaeorientalis*) serves as a critical indicator species reflecting the health of the Yangtze River ecosystem, China. Understanding its genetic diversity and population structure is essential for effective conservation strategies. We conducted a population genetics analysis of the Yangtze finless porpoises in Poyang Lake using two molecular markers: microsatellites and mitochondrial DNA D-*loop* sequences. The mitochondrial genetic diversity indices of the Poyang population were Hd = 0.481 ± 0.020 and Pi = 0.00078 ± 0.00030, while the microsatellite genetic diversity indices were Ho = 0.610 and He = 0.655. A moderate level of genetic differentiation was observed between the Poyang population and the Anqing population. Some of the deceased samples from the Anqing population may have originated in Poyang Lake or exhibited a migratory and gene exchange background between Poyang Lake and the Yangtze River. This research offers essential data for the development of future conservation strategies for Yangtze finless porpoises in Poyang Lake.

## 1. Introduction

**Figure 1:**
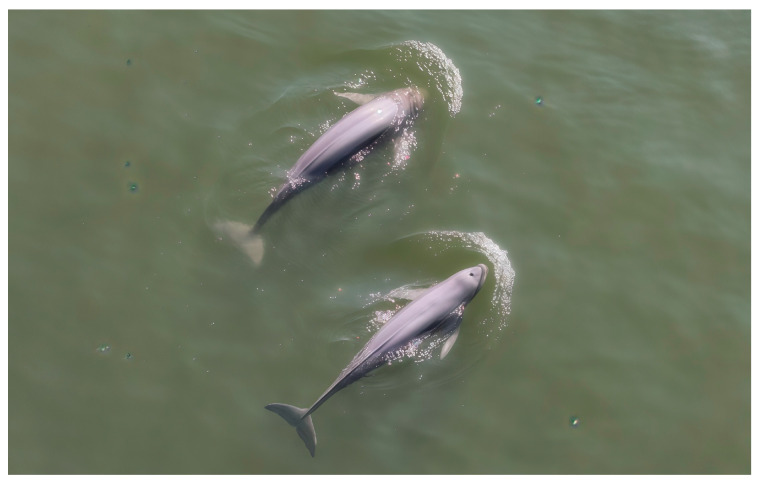
A picture of two Yangtze finless porpoises, *Neophocaena asiaeorientalis asiaeorientalis*, taken by Haiying Liang.

The Yangtze finless porpoise (*Neophocaena asiaeorientalis asiaeorientalis*; YFP) is a small, freshwater, toothed whale endemic to China (Figure 1). Currently, they primarily inhabit the middle and lower reaches of the Yangtze River, i.e., the section from Yichang to the Yangtze Estuary of the Yangtze River, as well as Dongting Lake, Poyang Lake, and its tributaries [1]. The rapid economic development along the Yangtze River section, coupled with the deteriorating ecological environment, has led to a sharp decline in the Yangtze finless porpoise population over the past few decades. Estimates show a decrease from approximately 2700 individuals in the early 1990s to slightly above 1000 at present [2]. In 2013, the YFP was designated as “Critically Endangered” (CR) on the IUCN Red List of Threatened Species. On 5 February 2021, the revised “List of National Key Protected Wild Animals” listed the YFP as a national first-class key protected wild animal [3,4]. As a crucial indicator species for the health of the Yangtze River ecosystem, YFPs remain endangered, necessitating an urgent and comprehensive conservation strategy [5].

Poyang Lake is an important aquatic life repository in the middle and lower reaches of the Yangtze River. The YFPs in Poyang Lake constitute approximately half of the total species population and serve as an important genetic resource pool for YFP conservation. Historically, there was a large-scale migration of YFPs at the junction of the main stream of the Yangtze River and Poyang Lake [2,3,6,7]. The confluence of Poyang Lake and the primary channel of the Yangtze River is a river segment frequently utilized by YFPs [8] and functions as an essential corridor for YFPs to maintain genetic exchange. Currently, studies have been conducted on the population dynamics, migration behavior, and community structure of YFPs in Poyang Lake. Liu et al. [9] determined that the YFPs in Poyang Lake have a seasonal distribution, with water depth being an important environmental variable affecting their distribution and habitat selection. Subsequent expeditions indicated that YFPs were prevalent throughout the main lake area of Poyang Lake and its principal tributaries (the Gan, Xin, Fu, and Rao Rivers). However, their distribution patterns exhibit seasonal variations. Fish resources and hydrological characteristics are hypothesized to significantly influence YPF distribution [10,11,12]. Acoustic monitoring data revealed that the YFPs in Poyang Lake are engaged in seasonal migratory activities between the main lake area and its major tributaries. During dry periods, the YFPs migrated from Poyang Lake to the Yangtze River, whereas during high water periods, they migrated from the Yangtze River to Poyang Lake [13]. Based on a kinship analysis of YFPs in Poyang Lake, Chen et al. [14] found that the YFP mating system is mixed. Specifically, the mother–offspring pair was identified as the most stable unit in the lake, whereas male YFPs exhibited random distribution throughout the lake area and appeared to have no involvement in nursery activities.

Evaluating the genetic diversity of threatened wildlife populations provides critical scientific knowledge necessary for implementing effective conservation measures and developing robust genetic management strategies [15,16,17]. The decline of global water ecosystems and human activities have placed numerous cetaceans at risk of extinction, including the Indo-Pacific humpback dolphin (*Sousa chinensis*), fin whale (*Balaenoptera physalus*), and humpback whale (*Megaptera novaeangliae*) [18,19,20]. The effective protection and reproduction of whales are crucial for maintaining biodiversity; therefore, further research on whale conservation genetics should be conducted in the future. The population genetics of the YFP, the sole surviving freshwater whale species in China, has been the subject of extensive discussion [21,22,23]. The continuous decline in YFP numbers and the emergence of YFP distribution gaps in recent decades raise concerns regarding potential losses in genetic diversity and the development of distinct genetic structures among the populations in different geographical regions of the Yangtze River. Initial analyses of the genetic diversity and structure of seven populations located in different sections of the middle and lower reaches of the Yangtze River indicated that YFPs had low genetic diversity. Significant genetic differentiation was found between the Xinchang–Shishou population in the middle reaches and five populations in different sections in the lower reaches of the Yangtze River [23]. These findings are corroborated by three distribution blank areas identified during the 2012 freshwater dolphin expedition in the Yichang-Shashi section of the Yangtze River, the Shishou–Yueyang section, and the 150 km river section upstream and downstream of Wuhan [24]. Notably, Chen et al. [25] performed a genetic analysis on 91 YFP samples collected from Poyang Lake between 2009 and 2011. Their findings revealed that the Poyang population exhibited low mitochondrial genetic diversity and medium microsatellite genetic diversity. Furthermore, considerable genetic differentiation was observed between the Poyang population and four distinct populations from different geographic regions in the middle and lower reaches of the Yangtze River. In addition, Wang et al. [26] used 166 YFP samples collected from Poyang Lake between 2009 and 2021 to assess the genetic diversity of the Poyang population, revealing a moderate level of microsatellite genetic diversity. Nevertheless, approximately 90% of the YFP samples obtained from Poyang Lake in previous studies were obtained between 2009 and 2015. The Anqing section of the Yangtze River is one of the highest densities of YFPs, and it is connected to Poyang Lake, making it one of the closest river sections to Poyang Lake. The junction of the Anqing section of the Yangtze River and Poyang Lake is the most important area where the YFP river–lake migration and gene exchange are most likely to occur. However, no studies have analyzed the genetic differentiation between the Anqing and Poyang populations. Considering the notable decrease in the river–lake migration behavior of the YFPs in Poyang Lake and the decline in habitat quality [24,27,28], it is necessary to realize the current status of genetic diversity in the Poyang population and its genetic differentiation from the distinct populations in different sections of the Yangtze River.

The regular migration and gene flow between the Poyang population and the main Yangtze River population are essential for maintaining the genetic diversity of the species. To assess the current genetic status of this endangered species, we employed mitochondrial D-*loop* sequencing and microsatellite analysis of samples collected during rescue operations in Poyang Lake. By integrating data from both live blood and death specimens from the Anqing section of the Yangtze River, we further investigated the genetic differentiation and potential connectivity between the Poyang and Anqing population. Our findings provide crucial insights for developing effective conservation strategies and ensuring the long-term viability of this ecologically significant cetacean species.

## 2. Materials and Methods

### 2.1. Animal Ethical Approval

We strictly followed the national animal welfare regulations and policy system in China. The medical examinations and related experiments complied with the Implementing Regulations of the People’s Republic of China on the Protection of Aquatic Wild Animals, promulgated in 1993 and revised in 2013. The physical examinations conducted, such as animal chasing, handling and blood collection procedures, and related experiments were approved by the Department of Resource Environmental Protection of the Yangtze River Basin Fisheries Supervision and Management Office of the Ministry of Agriculture (2017 [185]) and the Department of Agriculture and Rural Affairs of Jiangxi Province (Gan nong Zi [2022] 52 and 2022 [10]). Our study is reported in accordance with ARRIVE guidelines (https://arriveguidelines.org) (accessed on 19 August 2024).

### 2.2. Animals and Sample Collection

**Figure 2:**
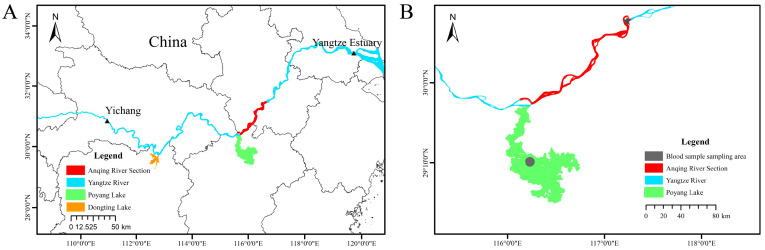
Distribution ranges of the YFP and sampling locations for the Poyang and Anqing populations. (**A**): The distribution range of the YFP in the middle and lower reaches of the Yangtze River extends from the Yichang section of the upper Yangtze River to the Yangtze River estuary (the area between the two black triangles), including Poyang Lake (green area) and Dongting Lake (orange area). (**B**): The sampling locations for the Poyang and Anqing populations in this study are indicated as follows: the green region represents the waters of Poyang Lake; the red region corresponds to the Anqing section of the Yangtze River. The gray circular area indicates the blood sample collection sites for the Poyang analysis, and the gray star-shaped area denotes the blood sample collection sites for the Anqing analysis. The red region signifies the collection sites for deceased tissue samples of the Anqing analysis.

A total of 171 YFP samples from the Poyang and Anqing populations were used in this study. The Poyang population contained 125 blood samples and the Anqing population contained 46 samples (21 fresh blood samples and 25 dead tissue samples). In 2017, the Freshwater Fisheries Research Centre of the Chinese Academy of Fisheries Sciences participated in the YFP ex situ conservation action (2017 [185]). During the census screening, blood samples were collected from 21 YFPs in the Anqing section of the Yangtze River. In addition, the Freshwater Fisheries Research Centre was commissioned by the Yangtze River section Fisheries Supervision and Management Office of the Ministry of Agriculture and Rural Affairs to collect and process YFP mortality samples from the lower reaches of the Yangtze River. Muscle samples were obtained from 25 YFPs that perished in the wild in the Anqing section of the Yangtze River between 2017 and 2023. Dead tissue samples were preserved via immersion in absolute ethanol at −20 °C. The sampling area and information for this study are presented in Figure 2 and Table 1, respectively.

In November 2022 and February 2023, in response to the severe threat posed by critically low water levels to the YFPs in Lake Poyang, we were commissioned by the Agricultural Rural Department of Jiangxi Province to participate in emergency rescue efforts. Fresh blood samples were collected from 125 YFPs, comprising 46 females and 79 males, with body lengths ranging from 92 to 165 cm. Fresh blood samples were collected from the tail veins of the animals using a 10 mL sterile disposable syringe. These samples were immediately placed in EDTA-K2 anticoagulant vacuum blood collection tubes and stored in an ultra-low temperature refrigerator at −80 °C after being transported to the laboratory at −20 °C prior to DNA extraction.

### 2.3. Sample DNA Extraction

DNA extraction was performed on fresh blood and dead tissue samples using the DNeasy Blood and Tissue Nucleic Acid Extraction Kit (QIAGEN GmbH, Hilden, Germany) according to the manufacturer’s instructions. The extracted DNA solution was stored at −20 °C and used as a template for subsequent polymerase chain reaction (PCR) amplification of the mitochondrial DNA control region (D-*loop*) and microsatellite DNA loci.

### 2.4. PCR Amplification and Sequencing

A 597 bp segment of the mtDNA control region was amplified using the forward (5′-GAATTCCCCGGTCTTGTAAACC-3′) and reverse primers (5′-GGTTTGGGCCTCTTTGAGAT-3′) [23] designed in our study with Primer Premier 5.0. The components of the PCR reaction system were as follows: 1 μL DNA template; 2.5 μL 10× buffer; 0.25 mmol/L dNTPs; 1 U Taq polymerase (Biostar, Toronto, ON, Canada); 1 μL each of primers (10 μmol/L); and the addition of ddH_2_O to a final volume of 25 μL. The PCR amplification program was as follows: the thermal cycling included initial denaturation at 95 °C for 5 min, followed by denaturation at 94 °C for 30 s, annealing at 60 °C for 30 s, and extension at 72 °C for 30 s. This cycle was repeated for 35 cycles, concluding with a final extension at 72 °C for 10 min. The amplification reaction was performed using an ETC-811 gene amplification PCR instrument (Dongsheng, Suzhou, China). The PCR products were tested by 1% agarose gel electrophoresis, followed by bidirectional sequencing of the successful products.

Drawing on the published articles [29,30,31,32,33], 20 pairs of polymorphic and steadily amplified microsatellite loci were selected for this study (Table 2). Each forward primer was labeled with the fluorescent dye 6-FAM at the 5′ end. The amplification reaction was performed using an ETC-811 gene amplification PCR instrument (Dongsheng, Suzhou, China). The PCR amplification reaction system comprised a total volume of 25 μL: 20–50 ng DNA template; 2.5 μL 10× buffer; 0.5 μL dNTPs (10 μmol/L); 1 U Taq polymerase (Biostar, Toronto, ON, Canada); 0.5 μL primers (10 μmol/L); and the remainder made up by adding ddH_2_O. The PCR amplification cycle parameters were initial denaturation at 95 °C for 5 min, followed by denaturation at 94 °C for 30 s, annealing at the annealing temperature for 30 s, and extension at 72 °C for 30 s. This cycle was repeated for 30 cycles, concluding with a final extension at 72 °C for 10 min. PCR products were separated by capillary electrophoresis using a denaturing acrylamide gel matrix on an ABI 3130XL automated sequencer (Applied Biosystems Inc., Foster City, CA, USA). The alleles were analyzed using GeneMapper v 5.0 (Applied Biosystems, Foster City, CA, USA) and visually checked according to the genotyping map.

### 2.5. Genetic Diversity Analysis

The mitochondrial DNA sequences from the Poyang and Anqing populations were analyzed using ClustalX V1.83 [34] to identify variant sites and determine haplotypes. MEGA V6.06 [35] was used to determine the base composition of the DNA sequences. The genetic diversity index of the two populations, including the number of haplotypes (H), nucleotide diversity (Pi), and haplotype diversity (Hd), was calculated using DnaSP V5.10.01 [36].

The microsatellite polymorphism indices for the Poyang and Anqing populations were calculated using GenAIEx6.51 [37], including observed heterozygosity (Ho), expected heterozygosity (He), and number of alleles (Na). FSTAT V2.9.4 was used to calculate the inbreeding coefficient (Fis) [38]. Cervus V3.0 was used to calculate the polymorphism information content (PIC) and conduct the Hardy–Weinberg equilibrium test for each seat [39].

### 2.6. Genetic Differentiation and Genetic Structure Analysis

We first performed genetic differentiation and gene flow analyses on 46 fresh blood and dead tissue samples from the Anqing population and 125 samples from the Poyang population. Considering the issue of untraceable dead tissue samples of the Anqing population, we performed genetic differentiation analyses again after removing 25 dead tissue samples from the Anqing population. Arliquin V3.0 [40] was used to test for significant differences, and the genetic differentiation coefficient (Fst) based on mitochondrial DNA was obtained by generating 10,000 random simulations. The analysis was performed using GenAIEx6.51 [37], and the parameters were set to calculate the F-statistic for determining the microsatellite-based genetic differentiation coefficient (Fst). Principal component analysis (PCA) was performed and plotted using the adegenet v.2.15 installation package [41] in R, and correlation analysis was performed using R 4.5.0 (http://www.r-project.org/) (accessed on 17 July 2024). The two-dimensional population structure distributions of the first and second principal components were plotted. A Bayesian model-based clustering analysis was conducted using STRUCTURE 2.3.1 [42] to infer the most likely number of genetic clusters (K). For this analysis, the admixture model and correlated allele frequencies were applied. For each K value (2–10), we performed 10 independent runs. Each run consisted of a burn-in period of 200,000 Markov chain Monte Carlo (MCMC) iterations followed by 1,000,000 MCMC iterations for data collection. The burn-in period and the number of replicates were consistent across all analyses. The most probable K value was estimated by calculating ΔK and mean LnP(K) using StructureSelector (https://lmme.ac.cn/StructureSelector/Contact.html#) (accessed on 18 July 2024). The gene flow (*Nm*) value was calculated using the following formula [43,44].

$$Nm=\left(1-Fst\right)Fst/4$$

## 3. Results

### 3.1. Genetic Diversity Analysis

In this study, 618 bp mitochondrial D-*loop* sequences were obtained from a total of 171 YFP samples, with no insertion/deletion sites detected in the aligned sequences. The A, T, G, and C contents of all sequences were 30%, 31.7%, 15%, and 23.3%, respectively, resulting in an A + T content of 61.7% and a G + C content of 38.3%, indicating a certain base composition bias. One mutation was detected in the 171 sample sequences, which defined two haplotypes (Hap1 and Hap2). The Poyang population (PY) mitochondrial genetic diversity indices were 0.481 ± 0.020 for haplotype diversity (Hd) and 0.00078 ± 0.00030 for nucleotide diversity (Pi), and the Anqing population (AQ1) mitochondrial genetic diversity indices were 0.496 ± 0.029 for haplotype diversity (Hd) and 0.00080 ± 0.00031 for nucleotide diversity (Pi). When only dead tissue samples were included, the mitochondrial genetic diversity indices of the Anqing population (AQ2) were 0.513 ± 0.037 for haplotype diversity (Hd) and 0.00080 ± 0.00041 for nucleotide diversity (Pi) (Table 3).

Using the 20 selected microsatellite primers, we detected 131 alleles (NA) in the 125 fresh blood samples of the Poyang population. The results showed that the number of alleles (Na) ranged from 4 to 12, with a mean of 6.55; the number of effective alleles (Ne) ranged from 1.417 to 5.919, with a mean of 3.436; the observed heterozygosity (Ho) ranged from 0.277 to 0.831, with a mean of 0. 610; the expected heterozygosity (He) ranged from 0.268 to 0.824, with a mean of 0.655; the PIC ranged from 0.285 to 0.809, with a mean of 0.622; and the inbreeding coefficient (Fis) ranged from −0.161 to 0.307, with a mean of 0.060 (Table 4). Except for Np404, Np428, and YFP69, the remaining 17 loci exhibited high levels of polymorphism, indicating that the selected microsatellite loci were highly polymorphic and contained a substantial amount of genetic information. The Hardy–Weinberg equilibrium (HWE) analysis showed that none of the 16 microsatellite loci deviated from the Hardy–Weinberg equilibrium (*p* > 0.05), except for Np409, PPHO130, and YFP59 at the SSR5 locus (*p* < 0.05).

Moreover, we detected 180 alleles (NA) in the 46 samples of the Anqing population. The results showed that the number of alleles (Na) ranged from 4 to 14, with a mean of 9; the number of effective alleles (Ne) ranged from 1.785 to 6.202, with a mean of 4.238; the observed heterozygosity (Ho) ranged from 0.326 to 0.844, with a mean of 0.623; the expected heterozygosity (He) ranged from 0.440 to 0.839, with a mean of 0.729; the PIC ranged from 0.408 to 0.819, with a mean of 0.696; and the inbreeding coefficient (Fis) ranged from −0.136 to 0.392, with a mean of 0.106 (Table 4). Except for Np404, Np428, and YFP69, the remaining 17 loci exhibited high levels of polymorphism, indicating that the selected microsatellite loci were highly polymorphic and contained a substantial amount of genetic information. The Hardy–Weinberg equilibrium (HWE) analysis showed that none of the 12 microsatellite loci deviated from the Hardy–Weinberg equilibrium (*p* > 0.05), except for Np409, Np428, PPHO130, YFP42, YFP69, YFPSSR40, YFPSSR41, and YFPSSR73 locus (*p* < 0.05). The results show that the genetic diversity level of the microsatellites in the Poyang population (Ho = 0.610, He = 0.655) is slightly lower than that in the Anqing population (Ho = 0.623, He = 0.729).

### 3.2. Genetic Differentiation and Genetic Structure Analysis

We combined 46 fresh blood and dead tissue samples of Anqing population to analyze the genetic differentiation between the Poyang and Anqing populations. The Fst values were 0.059 (mtDNA) and 0.0628 (SSR), while the gene flow (*Nm*) values were 3.99 (mtDNA) and 3.73 (SSR). Given the uncertainty in tracing the origins of the dead tissue samples, we excluded 25 such samples and performed an additional genetic differentiation analysis for the two populations. The resulting Fst values were 0.0732 (mtDNA) and 0.101 (SSR), respectively. The results indicated an increase in genetic differentiation between the two populations (Table 5).

**Figure 3:**
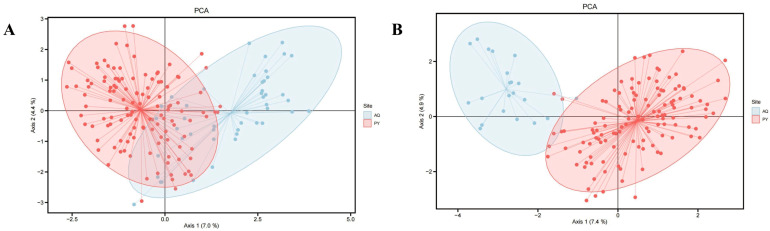
Results of Principal Component Analysis (PCA) for the Poyang and Anqing populations based on 20 microsatellite loci. (**A**): PCA results for the two populations before excluding dead tissue samples from the Anqing population. (**B**): PCA results for the two populations after excluding dead tissue samples from the Anqing population. The red area represents the Poyang population (PY), and the blue area represents the Anqing population (AQ).

**Figure 4:**
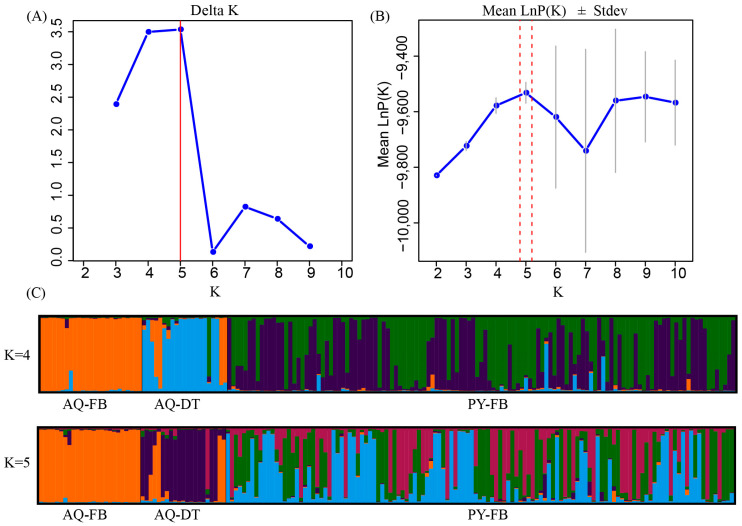
Bayesian analysis of the genetic structure of the Poyang and Anqing populations. (**A**): The corresponding Delta K values when K = 2–10. (**B**): The corresponding mean Ln P(K) values when K = 2–10. (**C**): The genetic clustering mixture model of all samples in the two populations when K = 4 and K = 5. Each individual is depicted as a vertical line partitioned into K segments, representing the admixture proportions derived from each genetic cluster. AQ-FB: All blood samples collected from the Anqing population. AQ-DT: All dead tissue samples collected from the Anqing population. PY-BF: All blood samples collected from the Poyang population.

The results of the PCA indicated that, in comparison to the clustering observed prior to the exclusion of the dead samples from the Anqing population (Figure 3), the clustering among individuals of the two populations became more dispersed following the removal of the dead tissue samples. This suggested an increase in the genetic differences between the two populations. STRUCTURE analysis indicated K = 5 as the optimal number of genetic clusters, supported by the maximum value of ΔK (Figure 4A) and the peak in mean LnP(K) (Figure 4B). This partitioning revealed distinct genetic clusters corresponding to the Anqing and Poyang populations. Moreover, some sample individuals in the AQ-DT cluster show genetic admixture with the Poyang population (Figure 4C).

## 4. Discussion

The effective conservation of genetic diversity is important for preventing inbreeding and loss of adaptation in endangered animals [45]. Genetic molecular markers can be used to detect genetic differences between the genes of different individuals, facilitating the understanding of variation within species populations and the level of genetic diversity [46]. Zhou et al. [29] investigated the relocated protection population of YFPs within the Tian’ezhou Baiji National Nature Reserve, which was affected by a severe snowstorm in the spring of 2008. Their evaluation revealed that, despite this event, the population maintained a moderate level of microsatellite genetic diversity (Ho = 0.574, He = 0.572). Chen et al. [25] analyzed haplotype detection in YFP samples collected from Poyang Lake between 2009 and 2011, identifying three haplotypes (Hap1, Hap2, and Hap8). The mitochondrial genetic diversity indices, Hd and Pi, were 0.52 and 0.009, respectively. In this study, we found that the genetic diversity indices obtained from the analysis of the Poyang population and the Anqing population were Ho = 0.610, He = 0.655 and Ho = 0.623, He = 0.729, respectively. In addition, compared with other cetacean species that share similar ecological habits, family structures, and migratory patterns, such as the Harbour porpoise *Phocoena Phocoena* (Ho = 0.470, He = 0.473) [47], the Indo-Pacific humpback dolphin *Sousa chinensis* (Ho = 0.497, He = 0.525) [48], and the endangered Franciscana *Pontoporia blainvillei* (Ho = 0.407, He = 0.452) [49], the YFP exhibits a relatively high level of microsatellite genetic diversity and has maintained relative stability over the past two decades. During the precipitous decline in YFP numbers, the genetic diversity of the principal populations did not exhibit a notable decline, suggesting that conserving the genetic diversity of YFPs is relatively optimistic.

Furthermore, the Fis value of the Poyang population in this study was 0.060, markedly higher than that of the Poyang population in 2009–2021 (Fis = 0.017) [26] and the Tian’ezhou ex situ conservation population in 2010 (Fis = 0.046) [50]. On the one hand, the relatively local sampling range of YFP samples in the Poyang population (Figure 1), which does not represent the entire Poyang Lake, along with numerous mother–child YFP pairs within this population, suggests a significant number of genetically related individuals in the Poyang population. On the other hand, this raises concerns regarding potential inbreeding risks within this population or indicates that inbreeding may have already occurred. Therefore, regular censuses of the Poyang population and individual exchange activities are recommended to gradually optimize its genetic structure, improve the level of genetic diversity, avoid the risk of inbreeding, and promote sustainable development of the Poyang population.

The mitochondrial genetic diversity indices for the Poyang population were as follows: Hd = 0.481 ± 0.020 and Pi = 0.00078 ± 0.00030. When the haplotype diversity index (Hd) is less than 0.5, and the nucleotide diversity index (Pi) is less than 0.005, the population has low mitochondrial genetic diversity [51]. In conclusion, the present Poyang population exhibited a low level of mitochondrial genetic diversity, lower than that observed in the Poyang population during 2009–2011 (Hd = 0.52, Pi = 0.0009). Compared to various other marine whales, such as the East Asian finless porpoise (*Neophocaena asiaeorientalis sunameri*) in the Yellow Sea (Hd = 0.71, Pi = 0.003) and Korean waters (Hd = 0.65, Pi = 0.0011) [52,53], *Phocoena Phocoena* (Hd = 0.93, Pi = 0.011), *Delphinus delphis* (Hd = 0.949, Pi = 0.018), and *Lagenorhynchus obscurus* (Hd = 0.97, Pi = 0.0163) [33,54,55], the mitochondrial genetic diversity of the YFPs in Poyang Lake was significantly lower. Furthermore, Chen et al. [25] performed a haplotype analysis on YFP samples collected from Poyang Lake during 2009–2011 and identified three distinct haplotypes (Hap1, Hap2, and Hap8). In contrast, we detected only two haplotypes (Hap1 and Hap2) in the 125 samples analyzed. In summary, considering these factors, the results of our study may be attributed to the matrilineal inheritance characteristics of mitochondrial DNA markers and the relatively concentrated sample source, which inadequately represents the genetic diversity status of the Poyang population. This phenomenon can be ascribed to the slower evolutionary rate of cetacean mitochondrial DNA relative to that of other mammals [56,57]. In addition, the YFP’s population decline may have reduced the genetic diversity of the Poyang population, but the drop from 3 to 2 mtDNA haplotypes could reflect limited historical data or sampling bias. Larger, spatiotemporally broad studies are needed to confirm whether this represents true diversity loss or methodological artifacts. Robust genetic monitoring is critical for accurate conservation assessment.

The habitat fragmentation of YFPs, influenced by both natural and anthropogenic factors, is increasingly exacerbating [58,59]. This phenomenon further impedes gene flow among YFPs and may result in increased genetic differentiation between distinct populations. This is not conducive to the protection of YFPs. According to Wright’s criteria [60], a moderate degree of genetic differentiation between Poyang and Anqing populations (0.05 < Fst < 0.15) existed, and a certain amount of gene flow (*Nm* > 1) was maintained. Several studies have indicated varying degrees of genetic differentiation among different populations of YFPs [23,25], as evidenced by the multiple blank distribution areas identified in the YFP investigation. In addition to the YFP, some marine species, such as the fin whale (*Balaenoptera physalus*) [61], Tuku dolphin (*Sotalia fluviatilis*) [62], and Irrawaddy dolphin (*Orcaella brevirostris*) [63], also encounter issues related to patchy distribution and significant genetic differentiation within populations. Therefore, more attention should be paid to the genetics of cetaceans and their populations in the future.

Limited gene flow is a key factor in population genetic differentiation, arising from long-term interactions among various ecological factors [64]. Theoretically, for YFPs with a generation time of eight years [65], short-term human activities are unlikely to result in significant genetic differentiation among different populations distributed in different geographical regions of the Yangtze River. The previous study has shown that the Tongling population, which is located in the Tongling section of the Yangtze River, has low genetic differentiation from the Poyang population (Fst < 0.05) [23]. The Anqing section of the Yangtze River is located upstream of the Tongling section, and its geographical location is closer to Poyang Lake. However, the results of this study show that there is already medium-level genetic differentiation between the Anqing and Poyang populations at present (0.05 < Fst < 0.15). The reasons for this may be as follows: (1) There has been a long-term deficiency in gene flow between the Poyang and Anqing populations. (2) The construction of a lake-mouth bridge across the lake and the significant increase in the number of navigable vessels have affected the natural rhythm of migratory movements of YFPs in the Poyang and Anqing populations. This has decreased the scale of migratory movements and subsequently hindered gene flow between the Poyang population and other different populations distributed in different geographical regions of the Yangtze River [3,5]. (3) In recent years, the recurrent low water levels in Poyang Lake have resulted in a dispersed distribution of YFPs within the lake and the five rivers connected to it, occasionally leading to seasonal isolation [66,67]. This phenomenon further affects the genetic exchange among YFP populations.

Furthermore, an increase in genetic differentiation (larger Fst values) was observed between the Poyang and Anqing populations based on mitochondrial DNA and microsatellites following the removal of 25 dead tissue samples from the Anqing population. Furthermore, STRUCTURE analysis revealed that some deceased individuals from the Anqing population exhibited relatively high genetic similarity to the Poyang population. Based on this, it is hypothesized that some dead tissue samples from the Anqing population may include individuals originating from Poyang Lake, indicating that some individuals that perished in Poyang Lake may have drifted downstream to the Anqing section of the Yangtze River, where they were subsequently discovered. Poyang Lake is connected to the Anqing section of the Yangtze River. The YFPs with the Yangtze River and Poyang Lake migration background may have been present in the dead tissue samples of the Anqing population, and their exclusion resulted in increased genetic differences between the two populations. The current absence of effective methods for accurately tracing the habitats of deceased YFPs complicates the determination of their origins. Future investigations should leverage strontium isotopes for this purpose. This approach represents a critical research avenue for enhancing YFP rescue efforts and habitat conservation strategies. We recognize that the use of both fresh blood samples and postmortem samples with uncertain origins may affect DNA quality and genotyping reliability. Although quality control measures were implemented (e.g., duplicate analyses, rigorous sample filtering), potential limitations remain, including (1) DNA degradation in postmortem samples and (2) unverified collection localities. These factors could potentially influence estimates of genetic diversity and haplotype detection accuracy. Future studies should adopt standardized sampling protocols and rigorously verify sample origins to enhance data quality. Such improvements will contribute to more accurate population genetic assessments, thereby supporting effective conservation management strategies.

## 5. Conclusions

The YFPs in Poyang Lake constitute approximately 50% of the species population. Poyang Lake is an important genetic resource base for YFPs. Considering the significant reduction in migratory behavior among aquatic environments and the degradation of habitat quality for YFPs in Poyang Lake, it is probable that the genetic diversity of this population may be adversely affected. We analyzed the genetic diversity and differentiation of the Poyang and Anqing populations. The genetic diversity analysis revealed two haplotypes in the Poyang population, with mitochondrial genetic diversity indices of Hd = 0.481 ± 0.020 and Pi = 0.00078 ± 0.00030. Additionally, microsatellite genetic diversity indices were recorded as Ho = 0.610 and He = 0.655. Furthermore, a moderate degree of genetic differentiation was observed between the Poyang and Anqing populations. This study provides essential data to support the scientific prediction of future trends in genetic diversity among different populations of Poyang Lake and the main channel of the Yangtze River while also guiding the development of conservation measures for YFPs.

## Figures and Tables

**Table 1:** Sampling information of Yangtze finless porpoise of the Poyang population (PY) and the Anqing population (AQ) in this study.

Population	PY	AQ	
Year	Number of Fresh Blood Samples	Number of Fresh Blood Samples	Number of Dead Tissue Samples
2017	0	21	5
2018	0	0	3
2019	0	0	2
2021	0	0	7
2022	105	0	2
2023	20	0	6
Total	125	21	25

**Table 2:** Twenty pairs of microsatellite loci selected for this study.

Locus	Primer Sequence (5′-3′)	Repeat Motif	Tm (°C)	Modification	Product Range/bp	Reference
YPFSSR5	F: GAGTGGGGTCAAATCAGGAA	(GT)_18_	60	5′ROX	206–226	Zhou et al., 2012 [29]
	R: ATGCCTTTGGCTGCATGTAT					
YFPSSR15	F: TGGAAAGAGGCCTTCAGATG	(GT)_22_CT(GT)_7_	60	5′6-FAM	177–217	Zhou et al., 2012 [29]
	R: TGACAGGTCCAAGAGCCAGT					
YFPSSR22	F: GCTCTCCTTGGCACTTTTCC	(AC)_10_	60	5′ROX	202–216	Zhou et al., 2012 [29]
	R: CCTCTCTGCCCAGTTTCCTA					
YFPSSR40	F: ATGAATTCTGTCCCCTGTGC	(AC)_16_	60	5′6-FAM	182–194	Zhou et al., 2012 [29]
	R: AGCCCAGTTATCTGGCTTCC					
YFPSSR41	F: TGACACAGGGAATTACTTTCAA	(GT)_16_	60	5′ROX	203–215	Zhou et al., 2012 [29]
	R: CCATGACCACGACAATAGCA					
YFPSSR51	F: TTAGTCAGCTCTCCCCATCC	(GT)_10_	60	5′TAMRA	199–209	Zhou et al., 2012 [29]
	R: TGCACACTCATACATGTACACACA					
YFPSSR73	F: TCCACCTGAGAAGCAAAACC	(TG)_22_	60	5′6-FAM	168–178	Zhou et al., 2012 [29]
	R: GGAACTGGCATTTAGGGTTG					
YFP1	F: TTTGGAAATTGCTAGACTGTGG	(AC)_15_	60	5′HEX	150–164	Zheng et al., 2008 [30]
	R: CCTCTTACGCAAGATAAAAGTGG					
YFP8	F: ATACTGGCAACAGCCACTAGGT	(AC)15	60	5′6-FAM	188–198	Zheng et al., 2008 [30]
	R: CACATTCTTTCCCTTTTTGTCC					
YFP42	F: TCCGTAGGCTTGGTTCTTGTAT	(GT)_11_(GA)_8_	60	5′HEX	166–184	Zheng et al., 2008 [30]
	R: AGGGGACCCTAAGTTTTCAGAG					
YFP59	F: GCACCTGGGTACTGTCCATATT	(CA)_15_	60	5′HEX	148–166	Zheng et al., 2008 [30]
	R: TCTTCCAAATACCTGCCTTCAT					
YFP69	F: GAGGACAGGGTGGTATGTTGTT	(GT)_14_	60	5′6-FAM	184–192	Zheng et al., 2008 [30]
	R: CATAGTCACCAGTGCATTTCCA					
YFPSSR63	F: ACCTGCCATAGCCCTCTTCT	(GT)_18_	60	5′TAMRA	192–202	Feng et al., 2009 [31]
	R: GTTTTGCGTGGAGTCAGACA					
YFPSSR71	F: GAAAAATGGGCTGTGTGGAT	(TG)_20_	60	5′TAMRA	190–200	Feng et al., 2009 [31]
	R: TGATTCAGTCACCAGCAACC					
YFPSSR75	F: GTTCATGGTTCCAGGGACTG	(AC)_14_	60	5′TAMRA	190–214	Feng et al., 2009 [31]
	R: CTCCCCAAATTCCCTTTTCT					
Np404	F: GGTCAGAACAAGAACACAG	(GATA)_3_GAT(GATA)_9_	60	5′HEX	160–168	Chen and Yang 2008 [32]
	R: CTCCTCCTAATACAGAAATAC					
Np409	F: TGGGAGAGGTATAAGTGGCT	(GATA)_3_GAT(GATA)_9_	60	5′ROX	205–257	Chen and Yang 2008 [32]
	R: TGGATGGGTGGAAGTAGTT					
Np428	F: CCAGAGAATCAGAACCAATAG	(GATA)_8_(GACA)_4_	60	5′HEX	113–133	Chen and Yang 2008 [32]
	R: CCAGAATCACACGAGCCT					
Np464	F: TGGCTGCACTTGCATTGATG	(GAAA)_5_A_2_(GAAA)_6_GA_2_G(GAAA)_5_	60	5′ROX	259–283	Chen and Yang 2008 [32]
	R: CCTAAGAACCCTCTAAATCCA					
PPHO130	F: CAAGCCCTTACACATATG	(CA)_25_	60	5′TAMRA	188–202	Rosel et al., 1999 [33]
	R: TATTGAGTAAAAGCAATTTTG					

**Table 3:** Genetic diversity parameters of the Poyang population and Anqing population as determined by mitochondrial DNA analysis.

Population	Distribution of Haplotypes		Haplotype Diversity	Nucleotide Diversity
	Hap1	Hap2	Hd	Pi
Poyang (PY)	76	49	0.481 ± 0.020	0.00078 ± 0.00030
Anqing (AQ1)	19	27	0.496 ± 0.029	0.00080 ± 0.00031
Anqing (AQ2)	11	14	0.513 ± 0.037	0.00080 ± 0.00041

**Table 4:** Genetic diversity indices of Poyang (PY) and Anqing (AQ) populations based on microsatellite loci.

				PY							AQ			
Locus	Na	Ne	Ho	He	PIC	Fis	HWE	Na	Ne	Ho	He	PIC	Fis	HWE
NP404	4	2.095	0.518	0.53	0.413	0.023	NS	4	2.115	0.489	0.527	0.469	0.012	NS
Np409	8	3.141	0.45	0.649	0.635	0.307	***	13	5.423	0.683	0.816	0.798	0.081	***
Np428	5	1.417	0.277	0.268	0.285	−0.035	NS	7	1.943	0.556	0.485	0.461	−0.136	NS
Np464	5	3.693	0.758	0.74	0.682	−0.025	NS	9	5.204	0.585	0.808	0.781	0.207	**
PPHO130	7	4.817	0.693	0.79	0.763	0.123	*	9	5.478	0.833	0.817	0.797	−0.085	NS
YFP1	6	3.424	0.782	0.714	0.667	−0.096	NS	6	2.95	0.667	0.661	0.601	−0.01	**
YFP8	12	3.896	0.831	0.715	0.712	−0.161	NS	6	4.268	0.644	0.766	0.724	0.113	*
YFP42	6	3.192	0.777	0.693	0.633	−0.121	NS	7	3.123	0.711	0.680	0.631	−0.032	NS
YFP59	8	5.919	0.731	0.81	0.809	0.098	***	10	6.202	0.778	0.839	0.819	0.041	**
YFP69	4	1.649	0.34	0.342	0.367	0.004	NS	7	1.785	0.326	0.440	0.408	0.270	NS
YFPSSR15	11	4.568	0.693	0.779	0.752	0.111	NS	11	4.594	0.558	0.782	0.751	0.267	***
YFPSSR22	4	2.519	0.582	0.596	0.541	0.023	NS	6	3.625	0.636	0.724	0.677	0.105	**
YFPSSR40	5	2.938	0.588	0.662	0.619	0.112	NS	9	5.414	0.844	0.815	0.792	−0.082	NS
YFPSSR41	8	5.659	0.623	0.824	0.802	0.245	NS	7	5.787	0.767	0.827	0.804	0.048	NS
YFPSSR51	5	2.946	0.72	0.662	0.608	−0.088	NS	6	4.029	0.614	0.752	0.713	0.133	*
YFPSSR63	3	2.409	0.5	0.585	0.502	0.146	NS	6	3.018	0.364	0.669	0.606	0.392	***
YFPSSR71	8	2.245	0.595	0.544	0.525	−0.094	NS	11	3.873	0.463	0.742	0.714	0.273	***
YFPSSR73	6	5.466	0.586	0.793	0.792	0.262	NS	11	5.781	0.783	0.827	0.804	0.046	NS
YFPSSR5	6	3.559	0.498	0.727	0.667	0.315	***	11	5.055	0.682	0.802	0.773	0.135	***
YFPSSR75	10	3.173	0.654	0.683	0.659	0.044	NS	14	5.095	0.477	0.804	0.788	0.335	***
Mean	6.55	3.436	0.61	0.655	0.622	0.060		9	4.238	0.623	0.729	0.696	0.106	

Note: PY: Poyang population; AQ: Anqing population. Number of alleles (Na); number of effective alleles (Ne); observed heterozygosity (Ho); expected heterozygosity (He); polymorphic information content (PIC); inbreeding coefficient (FIS); Hardy–Weinberg equilibrium (HWE): NS indicates no significant difference (*p* > 0.05), * *p*-value ≤ 0.05; ** *p*-value ≤ 0.01; *** *p*-value ≤ 0.001.

**Table 5:** The genetic differentiation coefficient (Fst) and gene flow (*Nm*) between the Poyang and Anqing populations, calculated before and after excluding the dead samples.

Molecular Marker	mtDNA	SSR
Before removing the dead samples	0.059 (3.99)	0.0628 (3.73)
After removing the dead samples	0.0732 (3.17)	0.101 (2.23)

Note: The values in parentheses are *Nm* values, and the values outside parentheses are Fst values.

## Data Availability

The mitochondrial D-*loop* sequences and specific microsatellite primers used in this study were obtained from published studies. Reference mtDNA sequences (Hap1, Hap2, Hap8) are available at Genbank (https://www.ncbi.nlm.nih.gov/genbank/) (accessed on 21 October 2023) (accession no. KC135874, KC135875, KC135881). The remaining data analyzed during this study are included in this published article.
